# Delayed Diagnosis of a Rare Case of Melorheostosis Affecting Both Upper Limbs

**DOI:** 10.7759/cureus.100281

**Published:** 2025-12-28

**Authors:** Md Nahiduzzamane Shazzad, Sharmin Nahar, Muhammad Shoaib Momen Majumder, Akash Ahmed Alif, Shamim Ahmed

**Affiliations:** 1 Rheumatology, Bangladesh Medical University, Dhaka, BGD; 2 Internal Medicine, Bangladesh College of Physicians and Surgeons, Dhaka, BGD

**Keywords:** candle wax, hyperostosis, melorheostosis, rare disease, skeletal dysplasia

## Abstract

Melorheostosis is an uncommon sclerosing bone dysplasia that primarily affects the long bones on one side, and occasionally hands or feet. The unique radiographic appearance, resembling dripping candle wax on the affected bone, is typically used to aid in diagnosis.

We present a case report of a 35-year-old woman who had had several bony swellings in her right hand, right forearm, right arm, left hand, and right upper chest for 28 years, which were slowly progressive. The patient complained of dull, throbbing pain and restricted joint movement in the affected areas. Plain radiography revealed flowing hyperostosis, resembling dripping candle wax, involving multiple bones of both upper limbs, including the right scapula and clavicle. We diagnosed her as a case of melorheostosis involving both upper limbs. She was treated with zoledronic acid, non-steroidal anti-inflammatory drugs (NSAIDs), duloxetine, pregabalin, and ongoing calcium and Vitamin D supplementation. Subsequent follow-up visits at 3, 6, and 12 months showed improvement in pain, with a halt to the progression of swelling.

## Introduction

Melorheostosis, also known as Leri disease, candle bone disease, or melting wax condition, is an uncommon chronic bone disorder. Leri and Joanny initially characterized it in 1922, originating from the Greek terms *melos* (meaning "limb"), *rhein* (meaning "flowing"), and *ostosis* (meaning "bone development") [1]. This condition is an unusual sclerosing bone dysplasia that is generally characterized by distinctive radiological features, such as hardened wax flowing down the edge of a candle [2]. It may mimic other sclerotic bone disorders, including osteoma, myositis ossificans, and parosteal osteosarcoma. Osteoma is a benign bone tumor that appears as a well-defined, radiopaque lesion on radiographs, in contrast to the irregular thickening of melorheostosis. Myositis ossificans presents as calcified masses within soft tissues following trauma, distinct from the smooth cortical thickening of melorheostosis. Parosteal osteosarcoma typically presents as a lobulated, dense mass with cortical disruption, in contrast to the uniform appearance of melorheostosis. The incidence of this condition is 0.9 per million, with no gender preference or inherited characteristics [3]. Recent studies have suggested that various somatic mutations, including MAP2K1, SMAD3, KRAS, and LEMD3, cause melorheostosis [4]. Patients may be affected at any age; however, the typical age of diagnosis is in early or late adulthood, often because of the absence of clinical symptoms. [5]. Any bone within the skeletal system may be impacted. The extremity bones are commonly affected, and involvement of the axial skeleton is rare [5]. The disease progresses chronically, with early-stage signs sometimes identified inadvertently or manifesting as pain, edema, deformities, contractures, limb length discrepancies, joint stiffness, and muscle atrophy [5]. The distribution is typically unilateral, with rare cases of bilateral involvement [6]. Despite the global prevalence of this condition, the literature search identified only one case from Bangladesh, indicating that it is under-diagnosed [7]. Diagnosis is typically established with plain radiography. Bone scintigraphy, CT, MRI, and bone biopsy may be required if X-ray results are unclear. The care is primarily symptomatic and is conducted by a multidisciplinary team, as there are currently no precise treatment guidelines.

## Case presentation

A 35-year-old woman presented with a 28-year history of progressive, occasional dull aching pain and bony swelling in her right upper extremity. The swelling initially involved the right middle and index fingers, subsequently extending to the right hand, forearm, and arm. Over the past two years, the swelling has progressed significantly, accompanied by persistent, dull pain that is not exacerbated by physical activity and is associated with restricted joint mobility. She observed comparable bony swelling in her left middle finger and the upper right chest. In this scenario, we initially considered different types of sclerosing bone disorders. She occasionally experienced difficulties carrying objects with her right hand. No pertinent trauma or family history was noted, although the patient's parents were in a consanguineous marriage.

**Figure 1:**
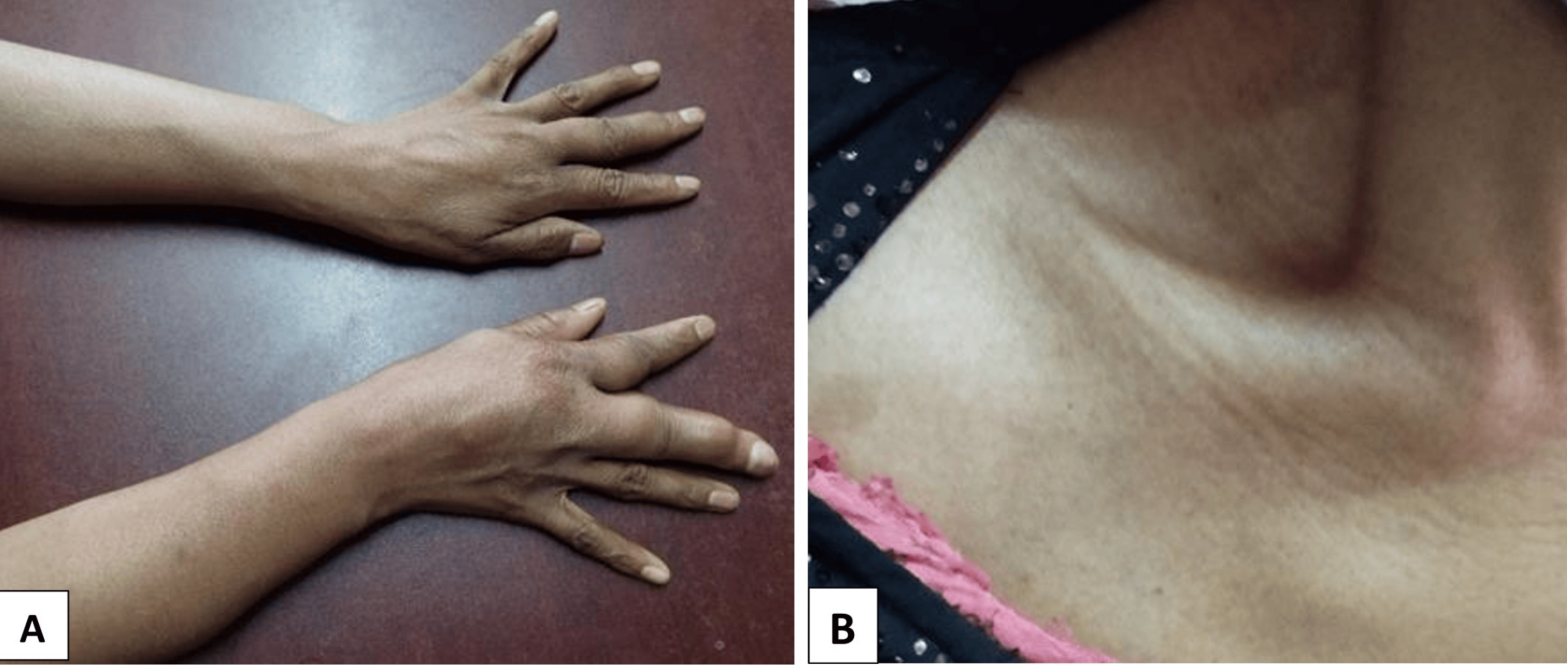
Image A displays swollen forearms and fingers; Image B shows swelling in the area over the coracoid process and clavicle.

Upon examination, the skin was soft, mobile and of normal thickness. The soft tissues of the involved areas were normal. The swelling was hard and non-tender, accompanied by a fixed flexion deformity in the right elbow joint and a fixed extension deformity in the right middle and index fingers (Figure 1).

**Figure 2:**
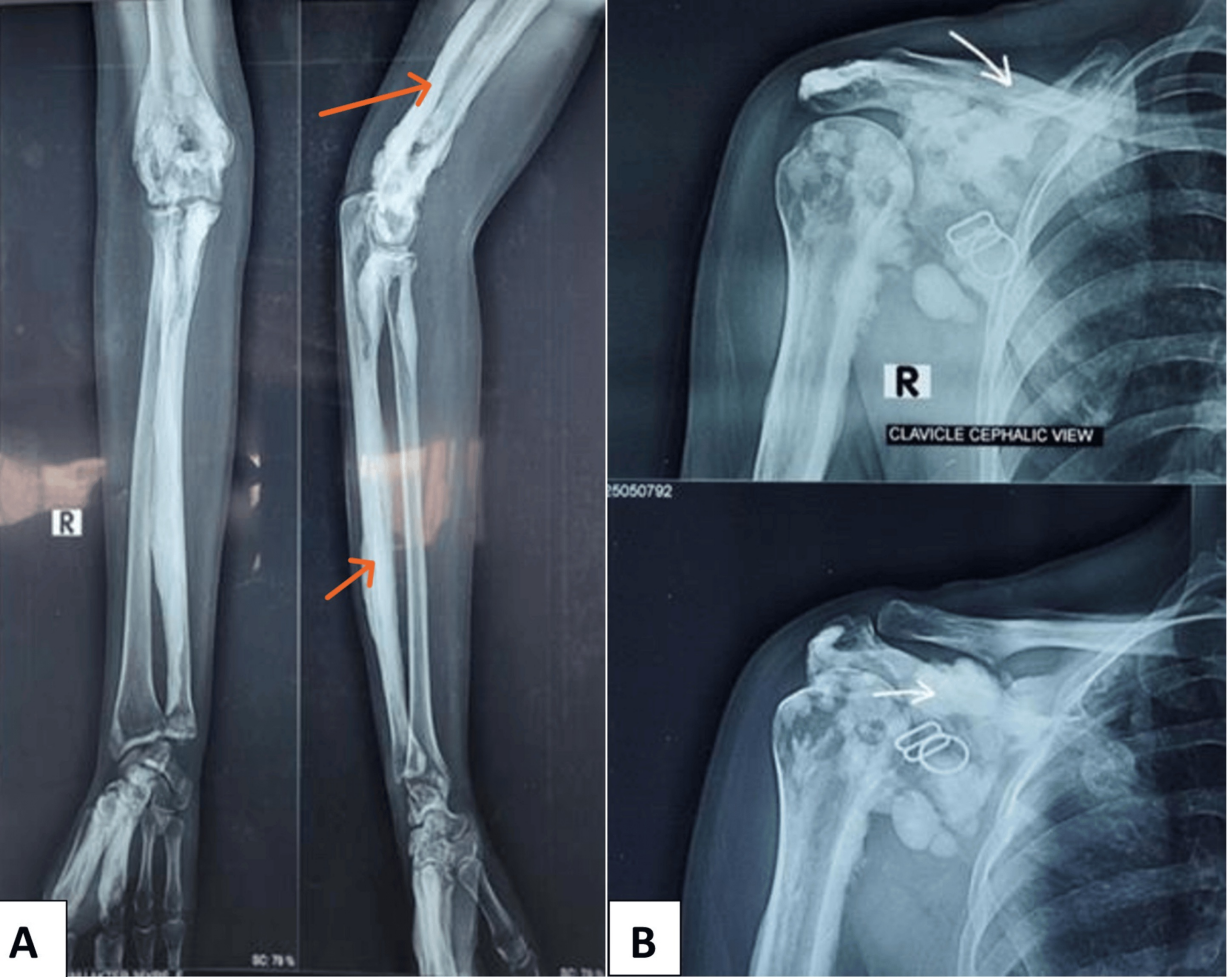
The X-rays show hyperostosis on the bones that looks like dripping candle wax, extending from the ulna to the lower humerus, and affecting the capitate, 2nd and 3rd metacarpal bones in Image A (orange arrows). Image B shows similar changes along the right clavicle, scapula, and humerus (white arrows).

Laboratory parameters, such as complete blood count, erythrocyte sedimentation rate (ESR), C-reative protein (CRP), rheumatoid factor, calcium, Vitamin D, and alkaline phosphatase, were within the normal range. Plain radiographs of the affected parts showed irregular cortical hyperostosis resembling candle wax, extending along the length of the affected bones (Figure 2). Upon the exclusion of alternative diagnoses, such as osteoma, myositis ossificans, and parosteal osteosarcoma, she was diagnosed with melorheostosis. She underwent treatment with zoledronic acid 5 mg annually, and received two doses. The patient was discharged with a regimen of naproxen 500 mg BD for 14 days, duloxetine 30 mg daily, and pregabalin 25 mg daily, along with calcium 750 mg daily and Vitamin D 1,000U daily supplementation. A three-monthly follow-up plan was scheduled. At follow-up visits at 3, 6, and 12 months post treatment, pain, discomfort, and range of motion improved. However, swelling remained unchanged.

## Discussion

Meleorheostosis is a rare sclerosing bone dysplasia characterized by cortical hyperostosis, typically affecting the long bones of the lower extremities [4]. Initially, it may be painless and less symptomatic. As the disease has a chronic course and is often asymptomatic in its early stages, it may lead to a delayed diagnosis, resulting in deformity [4]. It predominantly affects the long bones unilaterally, but rarely becomes bilateral [3,6]. The etiology of meleorheostosis remains unknown, although various theories have been proposed to explain it. Recent studies suggest that distinct somatic mutations, including MAP2K1, SMAD3, KRAS, and LEMD3, cause meleorheostosis [8].

In this case, the patient exhibited progressive swelling and pain in both upper extremities, consistent with polyostotic melorheostosis, which is less common [9]. To distinguish this condition from the other similar conditions described above, imaging modalities such as CT, MRI, bone scan, and biopsy may have been performed; however, because the plain X-ray showed a typical candle-wax appearance, the other imaging modalities were not obtained. A genetic study couldn't be performed due to unavailability.

The symptoms are consistent with the typical clinical manifestations of melorheostosis, including pain, swelling, deformities, contractures, and limb-length discrepancies [10]. The involvement of both appendicular skeletons in this patient is particularly significant, suggesting more extensive disease progression.

Radiographic imaging was essential in diagnosing meleorheostosis in this patient. The distinctive "dripping candle wax" image on radiographs is generally adequate for diagnosis. When radiographic data are ambiguous, additional imaging techniques, such as bone scintigraphy, CT, or MRI, may be required to confirm the diagnosis [11]. As our patient exhibited typical signs and symptoms, and the x-ray findings were typical, we didn’t pursue any further radiological evaluation.

Currently, there are no definitive treatment guidelines available due to the rarity of this condition and the limited research. Treatment was administered in accordance with prior case reports. The patient received zoledronic acid, a bisphosphonate recognized for its efficacy in reducing bone pain and decreasing bone turnover. Zoledronic acid has demonstrated substantial analgesic effects and improved quality of life in individuals with melorheostosis, despite not altering the fundamental disease process [12]. In addition to range-of-motion exercises for the affected part, appropriate doses of calcium and Vitamin D were administered.

The patient's follow-up at 3, 6, and 12 months indicated a reduction in pain; however, the bony swelling remained unchanged. This emphasizes the persistent nature of melorheostosis and the need for ongoing symptom management. The administration of non-steroidal anti-inflammatory drugs (NSAIDs), duloxetine, and pregabalin improved the pain in this patient.

## Conclusions

Slowly progressive bony enlargement with characteristic deformity, without significant pain, involving the long bones should raise suspicion of melorheostosis. Although a simple X-ray may provide an essential clue for early diagnosis, this patient's diagnosis was delayed. By reporting such a case, the medical community may be able to reach a quick diagnosis.
